# Evaluation and optimisation of a footwear assessment tool for use within a clinical environment

**DOI:** 10.1186/s13047-022-00519-6

**Published:** 2022-02-10

**Authors:** Stephen Ellis, Helen Branthwaite, Nachiappan Chockalingam

**Affiliations:** Centre for Biomechanics and Rehabilitation Technologies, Staffordshire University, Leek Road, ST4 2DF Stoke on Trent, UK

**Keywords:** Footwear, Shoes, Assessment, Validity

## Abstract

Footwear has been documented as a significant factor in the aetiology of foot pain in the general population. Assessing footwear in a clinical setting continues to be practitioner specific and there is limited guidance to direct advice. Health professionals must have access to clinically appropriate and reliable footwear assessment tools to educate patients on healthier footwear choices. The primary aim of this study was to critique what elements should be in a footwear assessment tool with a secondary aim of testing the agreed tool for validity.

A combined Nominal Group Technique and then a Delphi technique from purposively sampled experts of foot health professions were employed to critique elements of footwear assessment. The agreed tool was then tested by practising podiatrists on 5 different shoes to assess the validity and reliability of the measures.

Twelve test evaluation criteria were identified receiving significant ratings to form the final footwear assessment tool consisting of five footwear themes. Application of the tool in a clinical setting validated the themes of footwear characteristics, footwear structure, motion control and wear patterns. However, the assessment of footwear fit was not reliable.

The footwear tool was refined based on the collective consensus achieved from the rounds creating a more clinically appropriate tool. The validity of this tool was assessed as high in some of the themes but for those that were lower, a training need was identified.

**Supplementary Information:**

The online version contains supplementary material available at 10.1186/s13047-022-00519-6.

## Background

Footwear is an essential item of clothing to protect the foot from environmental damage yet a large proportion of the general population wear ill-fitting footwear [1], with a mismatch between the sizing of the shoe and the foot [2]. Wearing ill-fitting footwear can lead to clinical symptoms presenting, with up to 60% of foot pain in females due to ill-fitting footwear, with lack of depth of the shoe being a primary cause [3]. Additionally, ill-fitting footwear has been identified as a significant factor for falls in older adults [4], particularly when badly worn slippers without a fastening are worn, as the fit of the slipper can induce a trip [5]. Furthermore, footwear has been implicated as a major contributor in the development of diabetic ulcers with an estimated 74% of patients with diabetes wearing poor footwear which later led to foot amputation [6, 7]. It is therefore essential that footwear is discussed and examined at clinical appointments as part of a relevant assessment to prevent injury and improve foot health.

Evaluation of a patient’s footwear is often based on each individual’s needs, with attention to the choice of footwear worn and if the shoe is adequate for the purpose. Components of fit, wear and styling are often considered when assessing the choice of footwear made, particularly in relation to the presenting pathology [2, 7]. A useful way to start the conversation with a patient about footwear choice is with an assessment tool which not only needs to be simple, efficient and reliable but also suitable for use in a range of patient populations [8]. Designing a tool for specific patient groups allows for precise evaluation to be made associated with the needs of the presenting problem, as seen in diabetes [9] and falls [4]. These specific tools can be transferred into other patient groups [10] but by doing so may lose specific characteristics or not be relevant at all to the assessment. Measurements included in footwear tools need to be transferable to the proposed benefits extracted from the evaluation. In some cases, footwear assessment can be subjective and focus purely on the style rather than the suitability of the footwear [5]. Body image plays an important role in footwear choice [11] and should be included in the evaluation of footwear along with establishing a partnership between clinician and patient [12]. However, developing a reliable clinical record of footwear utilised by a patient remains challenging due to the complexity of previous tools and their relevance to clinical practice. This is evident in the tool developed by Barton et al., [8] where many components of the assessment include using equipment and measures not frequently observed in a clinical setting. Additionally, it is important to explore a range of footwear that a person wears in a variety of settings as they will not always attend a podiatry appointment in the shoes most often worn [13]. Therefore the tool needs to be focused and quick to use for multiple shoes to be assessed in one appointment.

This paper presents a new clinically focused footwear tool which has been created by debating the relevance of previously published footwear tools critiqued through expert rounds of consensus. This tool has then been validated by assessing its reliability as a method to consistently provide the same results, for its use by practising podiatrists. The aim of creating this tool is to provide clinicians with a clinical footwear assessment which is diverse in its application to ensure suitable advice is given. The purpose of this advice is to help patients choose shoes that are clinically relevant to the problems observed with fit and pathology and useability in mind.

## Methods

The initial phase was designed to test and evaluate existing parameters included in footwear tools using a Nominal Group Technique (NGT). Specifically, to review the efficacy, simplicity and practicality of current footwear assessment tools within a clinical environment. Following this, two rounds of consensus agreements using a modified Delphi methodology were implemented to further refine the criteria to include in a clinical assessment tool. On completion of these rounds, the new tool was produced and tested for reproducibility and repeatability measures to look for reliability and validity in a clinical environment.

Ethical approval was granted for each part of the research by Staffordshire University Ethics committee (RN0819LSE). At each part of the work, the participants were informed of the purpose of the phase and gave consent to take part in the research. At all times ethical codes of practice were followed.

### Nominal group technique

The NGT group were recruited from a broad scope of practicing podiatrists who were invited to a 3-hour group discussion. In this session, the footwear tool from Barton et al.,[8] was utilised as an initial framework to start the discussion of parameters used in footwear assessment tools. Each test, from this tool, was assessed on a Likert scale of 1-10 based on the following statements of agreement:

- Is this test simple and easy to understand?
- Would you regard this test as practical?
- Is the test accurate?
- Does the test have a clinical application?

Additional to the agreement scale, the participants were also asked if they knew of a more reliable/alternative tools and tests for that aspect of footwear assessment. These additional tests were recorded then discussed, and further ranking process took place for a final position. Each item was discussed by the participants in terms of the item’s importance to creating a clinical tool. This final data was then used to inform the discussions and ranking in the Delphi consensus.

### Delphi consensus

Two rounds of questionnaires, employing a modified Delphi technique, were then implemented based on the end point of the NGT. Round 1 targeted mixed healthcare professionals, known to specialise in footwear prescription and advice including both podiatrists and orthotists. Round 2 included a broader field of podiatrists and orthotists involved in general care to ensure a mixed viewpoint was obtained.

During the consensus exercise, the participants were asked to rate each test from the modified assessment tool created by the NGT round for simplicity, practicality, accuracy and for being clinically applicable with the following additional questions.

- Were the participants aware of each test?
- What did the participants think about the test or item?
- Did the participant understand the rationale behind the test?

At the completion of both rounds, the ranking and statements were collated to create a new footwear assessment tool.

### Clinical application

**Fig. 1:**
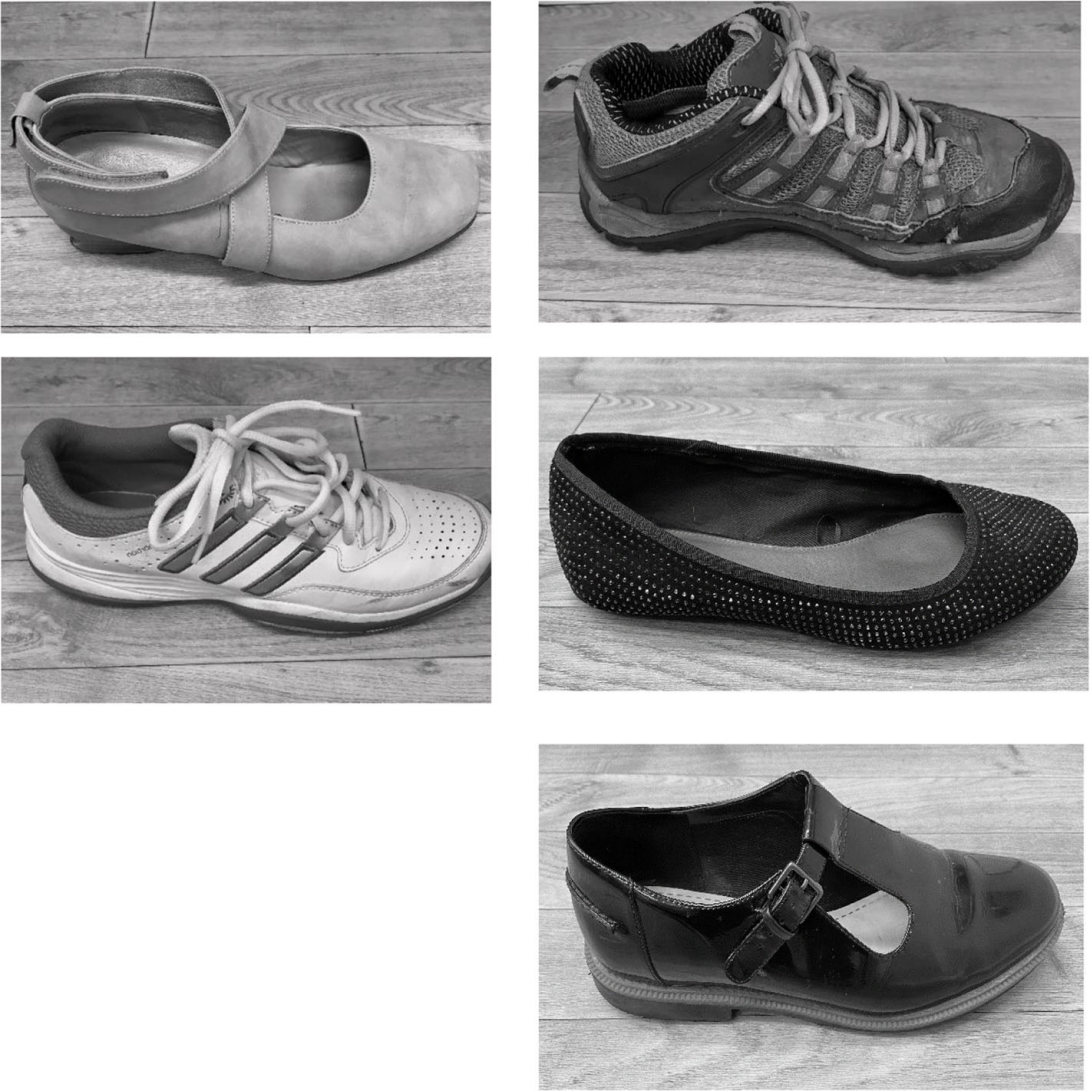
5 styles of footwear were chosen for clinical assessment. Each footwear style was different in size and structure to allow for application of the defined tool.

The new tool was then used to assess 5 different types of footwear on the same participant (Fig. 1). Four Health and Care Professions Council UK registered podiatrists, involved in providing a regular assessment of footwear, were recruited to assess all 5 items of footwear using the new tool. An information sheet was provided to the raters for reference to each test included in the tool to give clarity of wording used and tests to be performed. (Additional Files 1 and 2). Each podiatrist randomly assessed all 5 pairs at 3 different time frames with a gap of 3 weeks between each session.

### Analysis

For the 3 phases of data critique, the assessed ranking was calculated for every statement and converted into percentages agreements. Statements which ranked 70% of the maximum possible score were used to represent the agreement of the draft criteria [14] with anything below this value being disregarded. Additional to the rankings, open discussions were transcribed and coded manually into themes [Table 1]. From here a new clinical tool was created (Additional Files 1 and 2).

**Table 1:** Footwear measurements evaluated, defined as statements

Phases	One - Nominal Group Technique		Two and Three -Delphi
	Accepted statements with agreement above 70%	Rejected statements with agreement below 70%	Further rejection of statements with agreement below 70%
**Theme 1** Fit	1.1 **Removing the insole or shoe liner from footwear and comparing against foot and width (weight bearing).** • Draw around the foot (weight bearing) to obtain a cardboard template and compare to the inside of footwear width and length. 1.2 Plus 12 footwear measurement tool (objective measure).* 1.3 **Measurement of footwear depth and width (grasp test) subjective measure.**	2 Length A (rule of thumb) Subjective measure (subjective straw length measure). 3 Length B (Objective) measure, using Braddock type device, compared against a straw measure. 4 Place a tape measure around the foot (measure width and card between toe/s to measure the depth and height of the toe box).	5 Draw around the foot (weight bearing) to obtain a cardboard template and compare to the inside of footwear width and length.
**Theme 2** Footwear Characteristics	2.1 **Footwear style (using picture/photographs as examples).** • Using various footwear catalogues as examples of footwear. 2.2 **Materials (upper) different categories of materials.** 2.3 **Materials (outside) different categories of materials.**	• Age of shoe. • Age of Shoe using clinical judgement, due to variable factors such as the patients’ weight, frequency and amount of use. • Weight and length ratio (using scales and Brannock-style device).	• Using various footwear catalogues as examples of footwear.
**Theme 3** Footwear Structure	3.1 **Heel height (using a ruler).** 3.2 **Forefoot height measured (with a ruler) using 1st and 5th Metatarsal phalangeal joints as reference points.** 3.3 **Normalised longitudinal profile (heel – forefoot difference, or pitch).**	4 The last shape (measured by bisecting the heel and forefoot areas on the shoe sole. Then measuring the angular difference between the two using a goniometer. 5 Fixation of the upper sole. 6 Forefoot sole flexion point.	
**Theme 4** Motion Control Properties	4.1 **Fastening (various types).**	• Density (Single or Multiple). • Heel counter stiffness (subjective measure). • Scale for motion control properties. • Mid foot sole frontal stability (torsion). • Mid foot (or longitudinal) sole sagittal stability.	
**Theme 5** Cushioning		• The presence of cushioning system (Types and location within the footwear. • Lateral side hardness (Subjective measure). • Lateral midsole hardness (objective) using a penetrometer. • Medial midsole hardness (subjective). • Medial midsole hardness (objective) using a penetrometer). • Heel sole hardness (subjective). • Heel sole hardness (objective) using a penetrometer.	• Theme rejected as no statements reached agreement
**Theme 6** Wear Patterns	6.1 **Wear Patterns (Upper, midsole, tread pattern, and outsole).** • Upper (as above, however instead to) Semi quantitative or quantitatively describe the medial and lateral tilt. 6.2 **Tread Pattern (Types and amount of wear for the whole sole area).** 6.3 **Outer sole wear pattern. (Specific wear patterns).**	• Upper (Neutral, Medial tilt greater than 10°, or lateral tilt greater than 10°. • Midsole- (Neutral, medial or lateral tilt).	• Upper (as above, however instead to) Semi quantitative or quantitatively describe the medial and lateral tilt.

Those statements that reached a level of agreement above 70% were taken into the Delphi rounds. Text highlighted in bold were agreed at all levels of critique and therefore informed the footwear tool. *Plus 12 footwear tool is commercially available

The newly developed tool was used in the clinical assessment of 5 different shoes (Fig. 1). Each section of the tool was coded to enable comparable inter and intra rater agreement. Inter rater analysis examined the scores on the same day between each of the 4 raters. Intra rater analysis explored scores at initial use of the tool compared to 3 weeks later and also 6 weeks later for each individual rater. Data were separated from the tool as categorical (category assessment of the shoe) and continuous data (measures of foot and heel height). Categorical data were assessed using a percentage agreement between raters and time frames. Coding of the tool included defining areas of the shoe into zones, this was based on plantar pressure mapping [15]. Mean values were used to define reliability with a high level of agreement set at 0.8 [16, 17]. Consistency of continuous data was calculated using Intra class correlation coefficients (ICCs) two-way mixed absolute agreement, with chance corrected agreement set at a substantial level of agreement above 0.6, a medium level of agreement and almost perfect set at 0.8, a high level of agreement, statistical significance was set at 95% confidence *p*<0.05.

## Results

### Nominal group technique

This group consisted of 8 participants, who were practising podiatrists within the UK. Table one indicates which statements from discussions and ranking of the footwear tool reached levels of agreement and which did not. Measures that did not reach 70% consensus agreement were rejected on low consensus around accuracy, clinical relevance and practicality. New statements that were brought to the group included the use of plus 12 measurement footwear tool.

### Delphi

This group consisted of 9 participants in total, 5 podiatrists and 4 orthotists. All of the participants were at an advanced level of clinical practice providing regular footwear advice. This round created further discussions on the statements around footwear assessment. There were 4 more of the statements that did not reach the 70% agreement level, as well as Theme 5 on cushioning, therefore they were disregarded for discussions in round 3 (Table 1).

The second round included 6 clinicians (podiatrist: *n*=4; orthotist: *n*=2). Consensus increased with levels of agreement reaching 98% for fastening in Theme 4, Motion Control, and agreement remaining above 80% for all statements in Theme 3, Footwear Structure and Theme 1, Fit.

### Clinical application

Categorical data from the tool for sole material, fastening, wear marks on the sole zone 3 and upper showed high percentage agreements for each rater over time as well as between raters with some factors receiving 100% agreement. Grasp, wear marks on sole zone 1,2 4-6 and insole width showed lower agreement for each rater over time than between raters (Table 2).

**Table 2:** Inter and Intra-rater percentage of agreements

Component measured	Intra-rater			Inter-rater			
	Week 0	Week 3	Week 6	Pod 1	Pod 2	Pod 3	Pod 4
Grasp	0.3	0.5	0.5	0.7	**0.8**	0.7	0.5
Depth	0.3	0.5	0.6	0.7	**0.8**	0.7	0.5
Material Upper	0.6	0.6	0.7	**0.8**	0.7	0.7	**0.8**
Material sole	**0.8**	**0.9**	**0.9**	**1**	1	**1**	**1**
Fastening	**0.8**	**1**	**0.9**	**0.8**	1	**1**	**0.8**
Sole Zone 1 (medial heel)	0.4	0.4	0.4	**0.8**	0.7	0.6	**0.8**
Sole Zone 2 (lateral heel)	0.6	0.5	0.7	0.7	**0.8**	0.6	**1**
Sole Zone 3 (midfoot)	**0.9**	0.6	**0.9**	**1**	**1**	**1**	**1**
Sole Zone 4 (hallux)	0.6	0.6	0.6	0.7	0.7	0.7	**0.8**
Sole Zone 5 (2-5 metatarsals)	0.6	0.6	0.4	0.7	**0.8**	**0.8**	0.7
Sole Zone 6 (digits)	0.6	0.3	0.4	0.6	0.3	0.7	1
Upper Zone 1 (medial heel)	**0.8**	**0.9**	**0.9**	0.6	**1**	**1**	**1**
Upper Zone 2 (lateral heel)	**0.9**	**1**	**0.9**	0.8	**1**	**1**	**1**
Upper Zone 3 (midfoot)	**1**	**0.9**	**0.9**	0.8	**1**	**1**	**1**
Upper Zone 4 (hallux)	0.6	0.5	0.5	0.4	**0.8**	0.7	0.6
Upper Zone 5(2-5 metatarsals)	0.5	0.5	0.5	0.6	**0.8**	0.7	0.7
Upper Zone 6 (digits)	**0.8**	**1**	**0.9**	**0.8**	**1**	0.7	**1**
Insole Length	0.5	**0.8**	0.6	0.7	0.7	0.7	0.5
Insole Width	0.7	0.6	0.3	0.7	**1**	0.5	0.5

Values are reported on a scale of 0-1 where 1 =100% agreement, for each categorical component measured on the tool. Bold indicates a high level of agreement. Zones used for analysis are related to plantar pressure mapping [15]

Continuous data measures for heel height were significant at each time point and between raters with almost perfect correlations observed. Foot length and shoe length measures were the least agreeable with no correlation observed for each rater between the 3- week time frames and an indifferent relationship for shoe length being seen between the raters (Table 3).

**Table 3:** Inter and Intra-rater interclass coefficient correlations for continuous data components

Component measured	Intra-rater			Inter-rater			
	Week 0	Week 3	Week 6	Pod 1	Pod 2	Pod 3	Pod 4
Heel height	0.81*	0.84*	0.79*	0.97*	0.97*	0.95*	0.95*
Forefoot height	0.91*	0.51	0.59	0.92*	0.42	0.98*	0.96*
Shoe style	0.17	0.73*	0.42	0.98*	0.93*	0.96*	0.33
Foot length	0	0	0	1*	0.63	0.78*	0.94*
Shoe Length	0.07	0.04	0.04	0.7	0.88*	0.92*	-0.6

Values are reported on a scale of 0-1 where 1 =100% agreement, bold text and an asterisk indicate statistical significance was observed as 95% confidence *p*<0.05

## Discussion

Creating a new footwear tool (Additional file 1) allowed for clinical opinions to influence the content and provide a relevant and usable clinical footwear assessment tool. The new tool proved to be reliable and valid for 4 of the 5 themes and therefore can be utilised effectively in clinical practice as an essential part of footwear assessment and evaluation across many different patient groups.

### Theme 1 fit

Getting a good fit of an individual’s shoe is thought to be an important element to preventing footwear related problems [18]. The consensus rounds defined the assessment of grasping the shoe and evaluating the depth of the shoe to be an important measure of fit (Theme 1.3), yet these measures in practice were the least agreeable with poor relationships observed over time. Similarly, poor validity was seen in the measurement of foot length and shoe length, despite a mismatch between measurement of the foot and inside of shoe being deemed as a useful clinical skill to indicate a fit issue from the consensus rounds (Theme 1.1). Mismatch of shoe size to foot size has been identified as common in adults and children [2, 19] as has a lack of depth to a shoe [3], both parameters are associated with pathology and pain. Although it is agreeable that these components of footwear assessment are important, the need for training and understanding on how to complete the assessments may be warranted as different methods for measuring the shoe were adopted. The plus 12 tool was used in the measurement of the shoes and foot and was brought to the NGT as a new tool to be used (Theme 1.2). This device accounts for 12mm toe gap at the end of the shoe and has been previously used when evaluating diabetic footwear [20]. However, this tool is not widely used in clinical practice and the lack of use and understanding of this measure could have been the reason for poor correlations when exploring the validity of the tool.

### Theme 2 footwear characteristics

Along with the fit of the shoe, the characteristics of footwear focused on styling and materials. Both were found to be important in clinical assessment. Having catalogues to demonstrate suitable footwear was disregarded at the final round of consensus as there was disagreement around the interpretation of an individual’s style preference (Theme 2.1). This component of footwear advice and choice is however thought to play a role in habits of selection and image should be discussed when discussing footwear [11]. The raters were able to identify footwear style with a high level of reliability observed. This excellent reliability supports the use of the tool for multi-professional clinics where more than one podiatrist cares for a patient group. Having consistency in interpretation and recording aids communication and continuity of care for individuals allowing identity, image and style to be recorded successfully. Other characteristics of the shoe included identification of sole material (Theme 2.3) which showed strong inter and intra reliability. Understanding materials used within footwear assists in advice, particularly when considering the activity and health of the patient. Slips and falls have been attributed to sole material in older adults and modification of this part of the shoe may be critical in the care of the patient [21].

### Theme 3 footwear structure

Extending the assessment of footwear characteristics towards the structure of the shoe led the consensus rounds to focus on the heel height (Theme 3.1). A high heeled shoe is well established as being ill fitting and inappropriate for everyday use [22]. However, a heel less than 0.5 cm can reduce balance and it is recommended that a heel of between 1 and 4 cm is used to maintain stability [23–25]. Therefore, clinical assessment and evaluation of heel height, heel drop and sole thickness are applicable when giving advice. The podiatrists testing validity were able to measure and assess the footwear’s heel height with excellent inter and intra reliability securing this theme as a valid part of the assessment. This will allow for fluid conversations on footwear advice to occur when evaluating the style of shoe chosen.

### Theme 4 motion control

Motion control of footwear was deemed less important from the rounds of discussion compared to the focus on motion in the literature, with only a fastening (Theme 4.1) being deemed as clinically relevant. Motion control in running footwear has been thought to play an important role in foot function for many years [26, 27], with recent work exploring its use for reducing injuries [28]. However, there is limited evidence to suggest that running footwear could be used as an injury prevention intervention for foot pathology even though the concept of improved performance and energetics is heavily supported in the running world [29, 30]. The use of everyday shoes to improve performance and reduce pain in patient populations has yet to be explored with clinicians not warranting evaluation and reporting of motion control relevant to practice. The use of a fastening was felt to be important and was reliably reported on in the tool with excellent levels of agreement. A fastening on the shoe has been identified as one feature that makes the footwear adequate for diabetic patients to help prevent ulceration [31, 32] and support the rheumatoid foot in managing pain [33]. An understanding of how different types of fastenings can benefit a variety of conditions and patient groups when giving clinical advice would improve the suitability of the discussions between patient and clinician.

### Theme 5 cushioning

Like motion control, cushioning of running footwear has been a key focus when identifying injury risk and improving performance [34, 35] however, this information was not widely acknowledged as relevant to a clinical population when assessing footwear. This component of footwear did not form any consensus from the rounds of critique and therefore was not included in the tool. Despite this cushioning footwear is commonly used in clinical situations to reduce pain in rheumatoid arthritis [36], plantar heel pain [37] and fat pad atrophy [38]. However, the use of cushioning insoles appears to be a preferred option for clinical intervention in patients with diabetes [39] and for reducing metatarsal pressure [40] which may explain why the discussions about including this theme within the tool came to no consensus as clinicians may focus on insole material for cushioning rather than footwear itself.

### Theme 6 wear patterns

The final theme of assessing wear patterns on the sole and upper was accepted as a suitable clinical measure of the footwear in the rounds of discussion (Theme 6.1). Wear marks on footwear have been linked to a potential cause of injury [41] with the tread geometry playing a role in how a sole unit of the shoe will wear [42]. Having a clinical assessment and understanding of wear on the shoe could provide an insight on how the shoes worn function for an individual. Within this work, to enable comparisons of where on the shoe wear marks were identified, the shoe was segmented into zones [15]. From the analysis of this, there was mixed inter and intra reliability with some zones providing excellent agreement and others not, indicating that there is less reliability in using wear marks for assessment of foot function. However, this lack of validity should not disregard the usefulness of assessing wear marks on shoes. A more comprehensive understanding of wear marks on sole units and uppers has yet to be established, even though clinical relevance appears to be embedded into practice.

The process of critically reviewing an existing footwear tool [8] for clinical practice highlighted the need for a simple, useable, and clinically relevant tool. Although the validation of the tool proved to be statiscally acceptable in many of the themes there was an observed training need identified from the group of podiatrists who took part with indication that some parts of the assessment form were misunderstood. This may have limited some areas of the tools assessment benefits and should be considered when utilising the tool in clinical practice. Provision of continual professional development training with a focus on detailed footwear assessment would enrich a podiatrists clinical footwear assessment and then the advice given to patients. Similarly, consideration should be given around the assessment of cushioning in a shoe, as although this was not felt to be a theme that had an agreement the relevance of cushioning is evident in footwear advice [36–38]. Further work to create a clinically relevant assessment feature for analysis of cushioning could provide clinicians with a suitable measure when assessing footwear. Additionally, evaluation of this tool within a multidisciplinary setting would allow for a wider view of footwear choice and assessment to give guidance on clinical advice given.

## Conclusions

Assessment and evaluation of footwear are integral to clinical practice providing valuable advice for patients with associated foot pathologies. Developing a clinically relevant tool that is useable, valid and reliable assists clinicians in the provision of care. The reported tool created from this work can be utilised in different clinical populations to assess and evaluate footwear chosen by patients to attend clinical appointments. It is a valid tool to incorporate into footwear assessment and will provide both educational discussions for the patient and clinician around footwear choice and suitability as well as providing a record of footwear characteristics for the clinician.

## Supplementary information

**Additional file 1**

**Additional file 2**

## Data Availability

Please contact author for data requests.

## Abbreviations

NGT: Nominal Group Technique
ICC: Intra Class Correlation
