# Supplementary material for: Evaluation and optimisation of a footwear assessment tool for use within a clinical environment
Source: J Foot Ankle Res. 2022 Feb 10;15:12. doi: 10.1186/s13047-022-00519-6 (PMC8829975; doi:10.1186/s13047-022-00519-6)

**Clinical Footwear assessment tool.**

| **Fit of Footwear (Theme 1)-** Indicate Left and Right with L and R if difference is observed. | | | |
| --- | --- | --- | --- |
| **Width Grasp** | Adequacy of footwear width, by grasping of the upper over the metatarsal heads. | | |
|  | Wide  (*excessive bunching of the upper)* | Good fit  *(slight bunching of the upper)* | Narrow  *(tight, taught upper unable to be grasped).* |
| **Depth** | Consideration of the ability of the toes and joints to move freely, and the absence of any pressure on the dorsal aspect of the toes and nails. | | |
|  | Deep  *(toes can maximally extend and do not move upper)* | Good Fit  *(toes can extend and contact upper)* | Shallow  *(toes cannot move or are restricted)* |
| **Size Match** | If possible, remove the insole or shoe liner from the footwear.  Compare the size against the foot length and width whilst weightbearing, evaluate if the insole is a match to size of the foot.  If this is not possible use the Plus 12 measure tool to assess the match. Measure the shoe length and the foot length. | | |
| Insole length | Long | Good match | Short |
| Insole Width | Wide | Good match | Narrow |
| Plus 12 length, shoe | mm | Foot Length | mm |
|  | | | |

| **Footwear Characteristics (Theme 2)** | | | | |
| --- | --- | --- | --- | --- |
| **Style (circle)** | 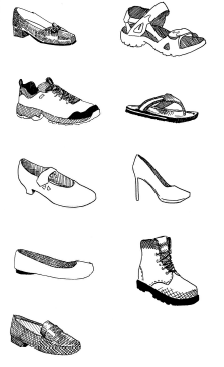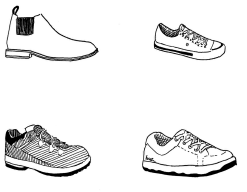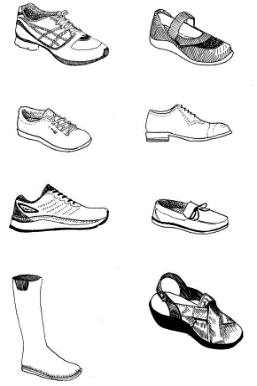 | | | |
| **Other style** |  | | | |
| **Materials Uppers** | The upper is commonly constructed from leather or synthetic materials and can be as individual components stitched together, one whole piece or knitted/woven | | | |
|  | Leather. | Synthetic, | | Mesh or knitted |
| **Other** |  | | | |
| **Materials Sole** | The sole of the shoe can be moulded onto the upper or is fixed as a separate component. | | | |
|  | Leather. | | Synthetic | |
| **Type of synthetic** |  | | | |
|  | | | | |

| **Footwear Structure (Theme 3 and 4)** | | | |
| --- | --- | --- | --- |
| **Heel Height** | Measurement recorded as the average of the height medially and laterally from the base of the heel to the centre of the heel-sole interface. | | |
|  | 0 to 2.5 cm | 2.6 to 5.0 cm | or > 5.0 cm. |
| **Forefoot height** | Measurement taken at the level of both the first and fifth metatarsal phalangeal joints and the average of both recorded. | | |
|  | 0 to 0.9 cm. | 1.0 to 2.0 cm | >2.0 cm. |
| **Drop or difference** |  | | |
| **Fastening**  **Theme 4** | Laces are considered the most optimal form of fixation as they allow the fit of the shoe to be individually adjusted, however, they can be difficult for some patients to manage. Other alternatives in these cases include straps/buckles, Velcro™, and zips. | | |
|  | Laces | Straps/buckles | Velcro™ |
| **Other** |  | | |
|  | | | |


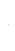


| **Wear Patterns (Theme 6)** | | | |
| --- | --- | --- | --- |
| Wear patterns of footwear can provide an insight into how an individual's foot is functioning in the shoe and may provide guidance as to when a shoe has become unsafe or requires replacement. Indicate Left and Right with L and R if required | | | |
| **Sole** | 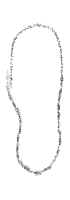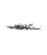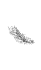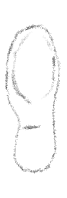 | **Upper** | 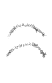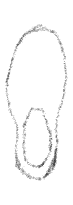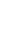 |
| - Tread - Wear position - Damage |  | - Scuff marks - Creasing - Damage |  |


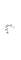

Supplement: Supplementary file 1 — Additional file 1 [file 13047_2022_519_MOESM1_ESM.docx]
