# Supplementary material for: Evaluation and optimisation of a footwear assessment tool for use within a clinical environment
Source: J Foot Ankle Res. 2022 Feb 10;15:12. doi: 10.1186/s13047-022-00519-6 (PMC8829975; doi:10.1186/s13047-022-00519-6)

**Clinical Footwear assessment tool.**

**INSTRUCTIONS.**

**There may be a need to highlight a left or right shoe measure. Indicate this on the form with a L for left and R for right when there is an observed difference.**

| **Fit of Footwear** | | | |
| --- | --- | --- | --- |
| **Width Grasp** | By grasping the upper over the metatarsal heads, as per picture. Evaluate whether the upper is too wide, a good fit or too narrow. Select your choice by circling the relevant option. | 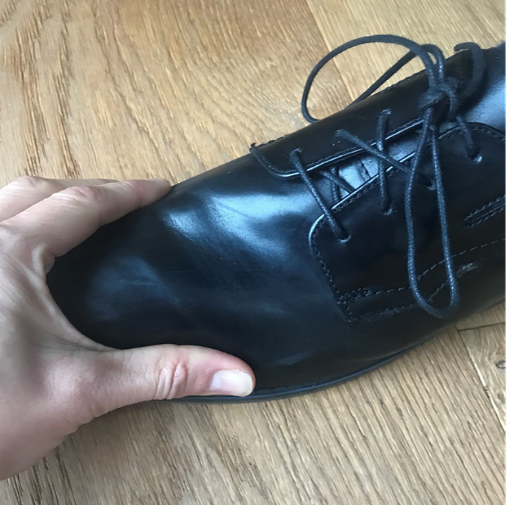 | |
| **Depth** | Consideration of the ability of the toes and joints to move freely, and the absence of any pressure on the dorsal aspect of the toes and nails. Evaluate how the toes move in the shoe and whether there is adequate room in the depth of the shoe. | 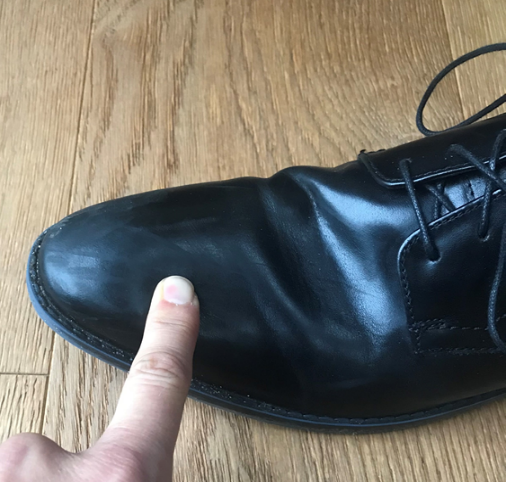 | |
| **Size match** | If possible, remove the insole or shoe liner from the footwear.  Compare the size against the foot length and width whilst weightbearing. Decide if the length and width of the insole match that of the foot and circle the appropriate choice.  If the insole does not come out or there is not an insole present, then use the plus 12 tool to distinguish if there is a difference in length and width of the foot compared to the shoe. | | |
| **Plus 12 measure** | Place plus 12 next to the foot and extend it so that the red tip is slightly longer than the toes. Let the tape measure stick out from under the longest toe. Read the millimetre value on the back of the measuring tape (this is the length of the foot including 12 mm clearance).  Then repeat this measure inside of the shoe. Record each measure | | 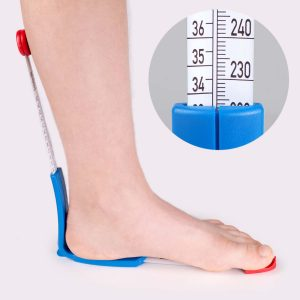 |
|  | | | |

| **Footwear Characteristics** | |
| --- | --- |
| **Style (circle)** | From the picture pick the style of shoe being tested, if there is no match describe the style of shoe in the other style box. |
| **Materials Uppers** | The main materials used in upper manufacture can be grouped into three main types. (Leather, synthetic or knitted mesh) If the material is something else then use the other box to describe – including if there is a combination of materials. |
| **Materials Sole** | The main choices of material sole are leather of synthetic. If the synthetic material is a mix (ie: EVA and PU) or similar, then use the Type of Synthetic box to record this. |
|  | |

| **Footwear Structure** | | |
| --- | --- | --- |
| **Heel Height** | Measurement recorded as the average of the height on the medial and lateral portion of the shoe from the base of the heel to the centre of the heel-sole interface. Measure both sides and average | 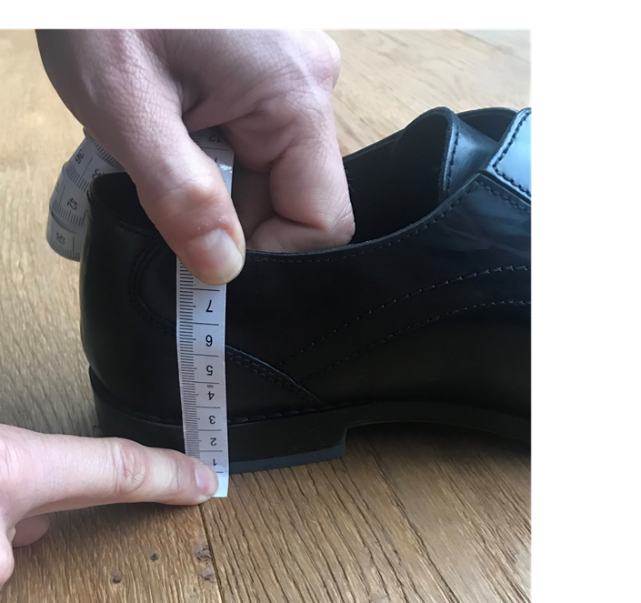 |
| **Forefoot height** | Measurement taken at the level of both the first and fifth metatarsal phalangeal joints and the average of both recorded. Measure both sides and average | 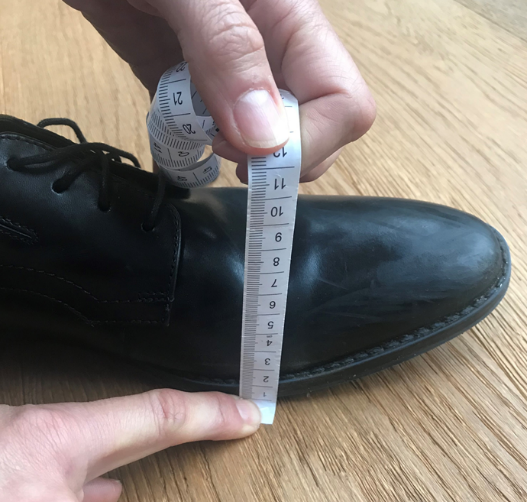 |
| **Drop or difference** | Subtract the forefoot height away from the heel height to get the drop of the shoe. Sometimes also referred to as the stack height. | |
| **Fastening** | Report the type of fastening on the shoe and if no fastening or another alternative from the list then comment on that in the other section. | |
|  | | |


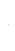


| **Wear Patterns** |
| --- |
| Wear patterns of footwear can provide an insight into how an individual's foot is functioning in the shoe and may provide guidance as to when a shoe has become unsafe or requires replacement. Use shading to indicate wear patterns on the sole of the shoes tread and any damage that has occurred. Use lines to show where scuff marks, creasing and damage has occurred on the upper of the shoe. |


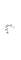

Supplement: Supplementary file 2 — Additional file 2 [file 13047_2022_519_MOESM2_ESM.docx]
